# Impact of changes in body mass index after percutaneous coronary intervention on long-term outcomes in patients with coronary artery disease

**DOI:** 10.1007/s00380-020-01648-3

**Published:** 2020-06-25

**Authors:** Hisanori Yui, Soichiro Ebisawa, Takashi Miura, Chie Nakamura, Shusaku Maruyama, Daisuke Kashiwagi, Ayumu Nagae, Takahiro Sakai, Tamon Kato, Tatsuya Saigusa, Ayako Okada, Hirohiko Motoki, Koichiro Kuwahara

**Affiliations:** 1 Department of Cardiovascular Medicine, Shinshu University School of Medicine, 3-1-1 Asahi, Matsumoto-shi, Nagano, 390-8621 Japan; 2 Department of Cardiology, Nagano Municipal Hospital, Nagano, Japan

**Keywords:** Obesity paradox, Percutaneous coronary intervention, Coronary artery disease, Prognosis

## Abstract

Little is known about the impact of changes in body mass index (BMI) after the percutaneous coronary intervention (PCI) on long-term outcomes in patients with coronary artery disease (CAD). Therefore, this study aimed to clarify this issue. We investigated data on CAD obtained from the SHINANO Registry, a prospective, observational, multicenter cohort study, from 2012 to 2013 in Nagano, Japan. One year after PCI, the enrolled patients were divided into the following three groups based on changes in BMI by tertiles: reduced, maintained, and elevated BMI. The associations among the groups and the 4-year outcomes [major adverse cardiac events (MACEs), all-cause death, Q-wave myocardial infarction, and stroke] were examined. Five hundred seventy-two patients were divided into the reduced, maintained, and elevated BMI groups. Over the 4-year follow-up period, the cumulative incidence of MACEs was 10.5% (60 cases). In the Kaplan–Meier analysis, the incidence rates of MACE were significantly higher in the reduced BMI group than in the maintained and elevated BMI groups [17.7% versus (vs.) 7.3% vs. 9.0%, *p* = 0.004]. Multivariable cox regression analysis showed that the reduced group showed increased risks of MACEs (hazard ratio 2.15; 95% confidence interval 1.29–3.57; *p* = 0.003). The long-term clinical outcomes of patients with CAD who underwent PCI were affected by the reduction in BMI after PCI. Furthermore, the elevation of BMI after PCI was not a poor prognostic factor.

## Introduction

Obesity is a poor prognostic factor in the general population [1], and being lean is an important determinant of premature death [2]. Obesity is generally assessed by body mass index (BMI), and individuals with obesity (BMI of 30.0 kg/m^2^) have a high incidence of coronary artery disease (CAD) [3]. A previous report demonstrated that obesity is an independent risk factor for developing heart failure [4]. However, weight loss has been reported to be an independent prognostic factor in patients who have already developed heart failure [5–7], and this is the “obesity paradox”.

Little is known about the impact of changes in BMI after PCI on long-term outcomes in patients with CAD. Weight changes after PCI can be used to provide patients with careful care, follow-up, and prognostic explanations if the existence of the obesity paradox in CAD is clarified. Therefore, this study aimed to clarify the impact of changes in BMI after PCI on long-term outcomes in patients with CAD.

## Methods

The SHINANO Registry was developed in accordance with the Declaration of Helsinki and was approved by the ethics committee of each participating hospital. Patients who gave written informed consent were enrolled.

The SHINANO Registry was a prospective, multicenter, observational, cohort registry of patients with any type of CAD, including stable angina, ST-segment elevation myocardial infarction (STEMI), non-STEMI, and unstable angina (UA), who underwent PCI in one of 14 hospitals in the Nagano prefecture of Japan between August 2012 and July 2013. This study was registered with the University Hospital Medical Information Network Clinical Trials Registry, as accepted by the International Committee of Medical Journal Editors (UMIN-ID: 000010070). This study had no exclusion criteria and was an all-comers registry.

**Fig. 1:**
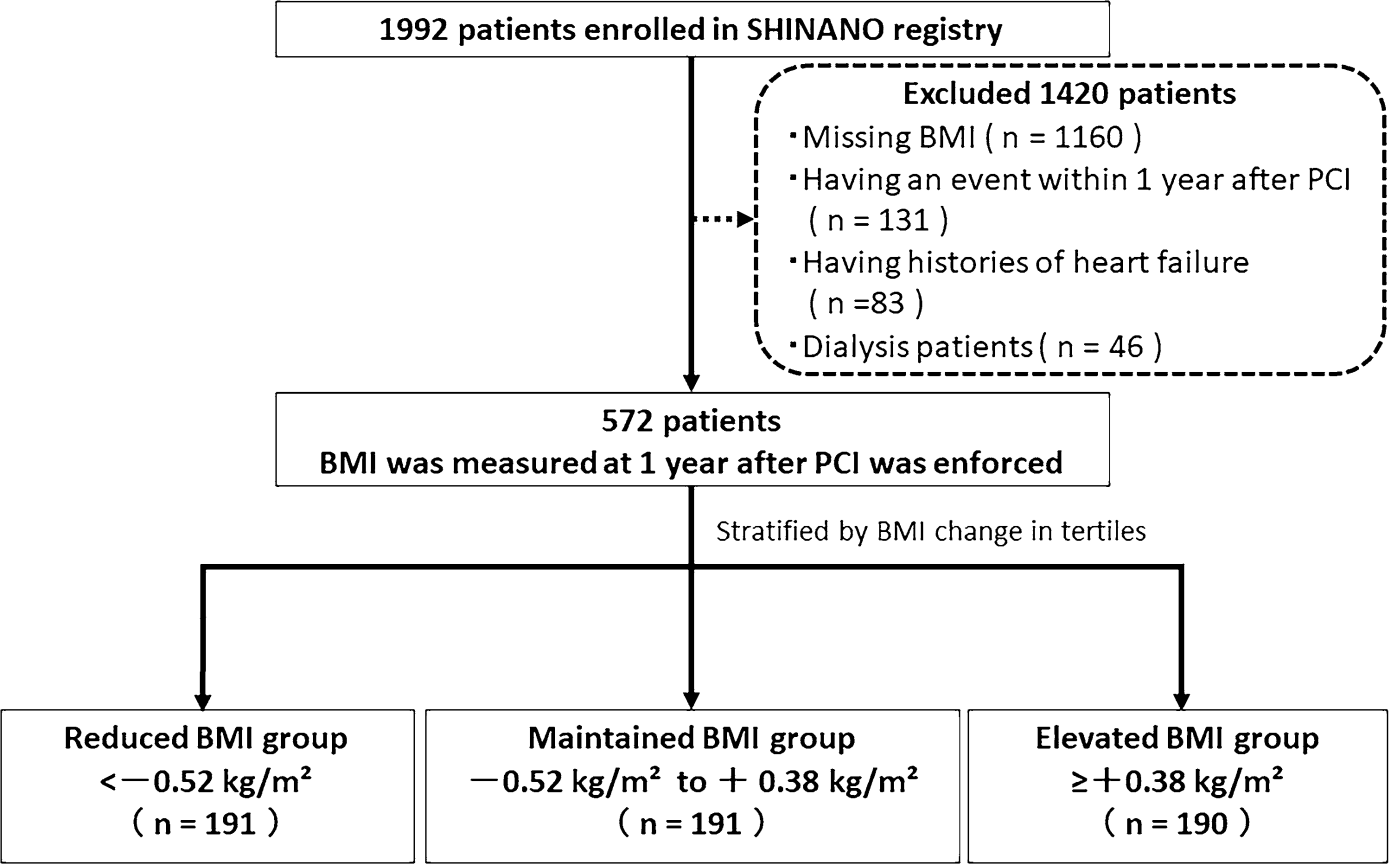
Study flow diagram illustrating the inclusion process and exclusion criteria. *BMI* body mass index, *PCI* percutaneous coronary intervention

The 1992 patients were registered in the SHINANO registry. We excluded 1420 patients with missing BMI (*n* = 1160), an event within 1 year after PCI (*n* = 131; because of the possibility that the condition was getting worse), and histories of heart failure and dialysis (*n* = 83 and 46; because of the possibility that the conditions affected weight). The time of initiating the follow-up period was from the point of 1 year after PCI. Patients were prospectively followed for 4 years. The final sample of 572 patients was divided into three groups based on the original amount of BMI change for 1 year in tertiles: reduced BMI group (< − 0.52 kg/m^2^; *n* = 191), maintained BMI group (− 0.52 to 0.38 kg/m^2^; *n* = 191), and elevated BMI group (≥ 0.38 kg/m^2^; *n* = 190) (Fig. 1).

The primary outcome was major adverse cardiovascular events (MACE), namely all-cause death, myocardial infarction (MI), and stroke at 4 years. The secondary outcomes were cardiac death, bleeding event, and target lesion revascularization. Cardiac death was defined as any of sudden death, death with no apparent cause other than heart disease, or death due to heart failure or myocardial infarction.

Bodyweight was measured to the nearest 0.1 kg using a digital scale, and height was measured to the nearest 0.1 cm; the patient stood without shoes. BMI was calculated by dividing weight (kg) by height squared (m^2^). Acute coronary syndrome (ACS) patients weighed on a weighable bed after PCI. ACS was a composite of STEMI, NSTEMI, and UA. ACS positive meant that the first PCI was performed on patients with ACS during the enrollment period. STEMI was diagnosed based on a chest symptom; ST elevation of 1 mV in two or more limb leads, or two contiguous precordial leads, or left bundle branch block; and elevation of biochemical markers of myocardial necrosis (troponin *T* value of 0.01 ng/mL or creatine phosphokinase level more than twice the normal range). NSTEMI was diagnosed based on a chest symptom, ST depression of 0.05 mV, *T* wave inversion of > 0.3 mV or transient ST-segment elevation of 0.05 mV, and elevated biochemical markers of myocardial necrosis. UA was diagnosed based on persistent resting or nocturnal chest pain with additional findings. Angiographic success was defined as the achievement of a reduction in the minimum stenosis diameter to 20% with grade 3 Thrombolysis in Myocardial Infarction flow. Diabetes was defined as a glycated hemoglobin level of ≥ 6.5%, fasting plasma glucose level of ≥ 126 mg/dL, or treatment with hypoglycemic agents. Hypertension was defined as a systolic blood pressure of ≥ 140 mmHg, diastolic blood pressure of ≥ 90 mmHg, or ongoing treatment for hypertension. Dyslipidemia was defined as a serum total cholesterol level of ≥ 220 mg/dL, a low-density lipoprotein cholesterol level of ≥ 140 mg/dL or higher, or receipt of current lipid-lowering therapy. Left ventricular ejection fraction (LVEF) was determined using the Teichholz method, and LVEF of ≤ 40% indicated left ventricular systolic dysfunction. Low BMI and high BMI were defined as BMI of < 18.5 kg/m^2^ and of > 30 kg/m^2^. A bleeding event was defined as a bleed that required treatment.

Normality was assessed according to results from a Shapiro–Wilk test. Continuous variables were reported as mean ± standard deviation if normally distributed as median and interquartile range otherwise. Categorical variables were reported as numbers and percentages. Group differences were analyzed using the *χ*^2^ test for categorical variables and the Kruskal–Wallis test for continuous variables. The Kaplan–Meier test was used to assess the cumulative incidence of MACE. The log-rank test was used to estimate group differences. Multivariable Cox regression analysis was used to identify the independent predictors of MACE; variables clinically associated with MACE were entered into the multivariable model. Analyses were performed using Statistical Package for Social Science version 25 (SPSS Inc., Chicago, Illinois, USA).

## Results

Baseline characteristics are shown in Table 1. The average patient age was 70.2 years, 77.2% were men, and the average BMI was 23.9 kg/m^2^ (range 14.1–38.8 kg/m^2^). Of the traditional coronary risk factors, 75.2% of patients had hypertension, 61.7% had dyslipidemia, 35.8% had diabetes mellitus, and 56.2% had a history of smoking. There was no significant difference in the background of the 3 groups. 1 year after PCI, the mean BMI was also in the normal range in each group: reduced BMI group (22.6 ± 3.7 kg/m^2^), maintained BMI group (23.8 ± 3.4 kg/m^2^), and elevated BMI group (24.8 ± 3.6 kg/m^2^). During the follow-up period, 60 cases (10.5%) of MACEs occurred, including 43 all-cause deaths, 4 non-fatal MIs, and 17 strokes (Table 2).

**Table 1:** Baseline patient characteristics

	Overall *N* = 572	Reduced BMI *N* = 191	Maintained BMI *N* = 191	Elevated BMI *N* = 190	*p* value
Age, years	71.0 (64.0, 77.0)	72.5 (64.0, 79.0)	70.0 (64.0, 77.0)	71.0 (64.0, 75.5)	0.36
Sex, no. of men (%)	442 (77.2%)	152 (79.6%)	148 (77.5%)	142 (74.7%)	0.53
BMI (kg/m^2^)	23.6 (21.9, 25.8)	24.1 (22.0, 26.5)	23.7 (22.0, 25.9)	23.3 (21.4, 25.1)	0.08
High BMI, *n* (%)	27 (4.7%)	12 (6.3%)	8 (4.2%)	7 (3.7%)	0.45
Low BMI, *n* (%)	27 (4.7%)	6 (3.1%)	9 (4.7%)	12 (6.3%)	0.34
Hypertension, *n* (%)	430 (75.2%)	147 (77.0%)	148 (77.5%)	135 (71.1%)	0.27
Dyslipidemia, *n* (%)	353 (61.7%)	118 (61.8%)	128 (67.0%)	107 (56.3%)	0.10
Diabetes mellitus, *n* (%)	205 (35.8%)	73 (38.4%)	74 (38.7%)	58 (30.5%)	0.17
Smoking, *n* (%)	322 (56.2%)	110 (57.6%)	108 (57.1%)	104 (55.6%)	0.92
LDL cholesterol level (mg/dL)	103.0 (84.0, 127.0)	104.5 (87.0, 122.0)	102.0 (81.0, 128.3)	106.0 (83.0, 129.0)	0.81
HDL cholesterol level (mg/dL)	47.0 (40.0, 56.0)	45.0 (39.0, 56.0)	47.0 (40.0, 55.0)	47.0 (41.0, 57.0)	0.46
TG level (mg/dL)	115.0 (82.5, 172.5)	114.5 (74.0, 180.0)	113.0 (83.8, 165.5)	118.0 (85.5, 174.0)	0.33
HbA1c level (%)	5.9 (5.6, 6.5)	6.0 (5.6, 6.6)	6.0 (5.6, 6.5)	5.9 (5.5, 6.3)	0.49
eGFR, (mL/min/1.73 m^2^)	65.2 (54.9, 78.8)	65.5 (54.3, 74.4)	67.3 (57.8, 81.3)	62.5 (51.0, 79.5)	0.20
LVD, *n* (%)	22 (11.7%)	22 (11.7%)	17 (9.0%)	16 (8.8%)	0.57
EF (%)	64.0 (55.0, 68.5)	65.0 (56.0, 68.0)	64.0 (54.9, 69.0)	62.7 (53.0, 68.0)	0.49
Af, *n* (%)	51 (8.9%)	15 (7.9%)	19 (9.9%)	17 (8.9%)	0.77
Aspirin use, *n* (%)	556 (97.2%)	187 (98.4%)	185 (97.9%)	184 (97.9%)	0.91
Thienopiridines use, *n* (%)	520 (90.9%)	171 (90.0%)	174 (92.1%)	175 (93.1%)	0.54
Warfarin use, *n* (%)	57 (10.0%)	16 (8.4%)	17 (9.0%)	24 (12.8%)	0.31
Statins use, *n* (%)	479 (83.7%)	159 (83.7%)	156 (83.0%)	164 (87.2%)	0.47
ACEi use, *n* (%)	201 (35.1%)	67 (35.3%)	66 (34.9%)	68 (36.4%)	0.95
ARB use, *n* (%)	209 (36.5%)	66 (34.7%)	73 (38.8%)	70 (37.4%)	0.70
Β-blocker use, *n* (%)	241 (42.1%)	71 (37.4%)	83 (44.4%)	87 (46.5%)	0.17
No. of diseased vessels	1.0 (1.0, 2.0)	1.0 (1.0, 2.0)	1.0 (1.0, 2.0)	1.0 (1.0, 2.0)	0.80
SYNTAX score	11.0 (6.3, 18.3)	12.0 (6.8, 18.0)	11.0 (6.8, 19.1)	10.5 (6.0, 19.0)	0.85
ACS, *n* (%)	259 (45.3%)	91 (47.6%)	75 (39.3%)	93 (48.9%)	0.12

Data are shown as median and interquartile range otherwise or *n* (%)

*ACE-I* angiotensin-converting enzyme inhibitor, *ACS* acute coronary syndrome, *Af* atrial fibrillation, *ARB* angiotensin receptor blocker, *BMI* body mass index, *EF* ejection fraction, *eGFR* estimated glomerular filtration rate, *HbA1C* glycated hemoglobin, *HDL* high-density lipoprotein cholesterol, *High BMI* high body mass index (> 30 kg/m^2^), *LDL-C* low-density lipoprotein cholesterol, *Low BMI* low body mass index (< 18.5 kg/m^2^), *LVD* left ventricular dysfunction (ejection fraction < 40%), *TG* triglyceride, *no.* number

**Table 2:** 4-year outcomes during the follow-up period

	Overall *N* = 572	Reduced BMI *N* = 191	Maintained BMI *N* = 191	Elevated BMI *N* = 190	*p* value
MACEs, *n* (%)	60 (10.5%)	31 (16.2%)	13 (6.8%)	16 (8.4%)	< 0.01
All-cause death, *n* (%)	43 (7.5%)	23 (12.0%)	9 (4.7%)	11 (5.8%)	0.01
Q-wave myocardial infarction, *n* (%)	4 (0.7%)	4 (2.1%)	0 (0.0%)	0 (0.0%)	0.02
Stroke, *n* (%)	17 (3.0%)	6 (3.1%)	5 (2.6%)	6 (3.2%)	0.94
Cardiac death, *n* (%)	38 (6.6%)	20 (10.5%)	8 (4.2%)	10 (5.3%)	0.03
Bleeding event, *n* (%)	23 (4.0%)	12 (6.3%)	6 (3.1%)	5 (2.6%)	0.15
Target lesion revascularization, *n* (%)	31 (5.4%)	13 (6.8%)	12 (6.3%)	6 (3.2%)	0.24

*BMI* body mass index, *MACE* major adverse cardiac or cerebrovascular events

**Fig. 2:**
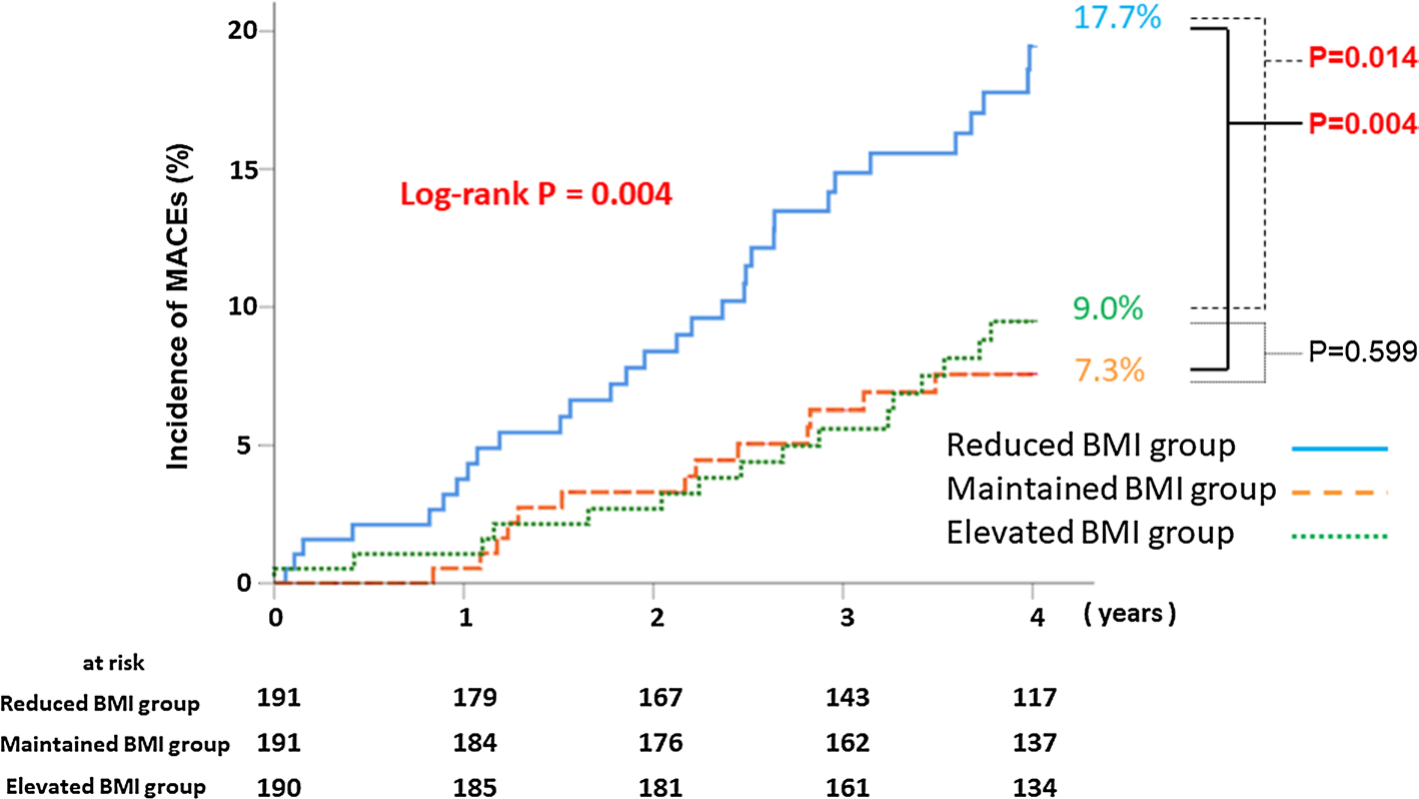
Kaplan–Meier analysis for the incidence of MACEs. Incidence of MACEs. The Kaplan–Meier curve for the cumulative incidence of MACEs is shown. MACEs include all-cause mortality, myocardial infarction, and stroke. The reduced BMI group (< − 0.52 kg/m^2^; *n* = 191) has a significantly higher incidence of MACEs than the maintained BMI group (− 0.52 to 0.38 kg/m^2^; *n* = 191) and elevated BMI group (≥ + 0.38 kg/m^2^; *n* = 190). *BMI* body mass index, *MACE* major adverse cardiac events

**Fig. 3:**
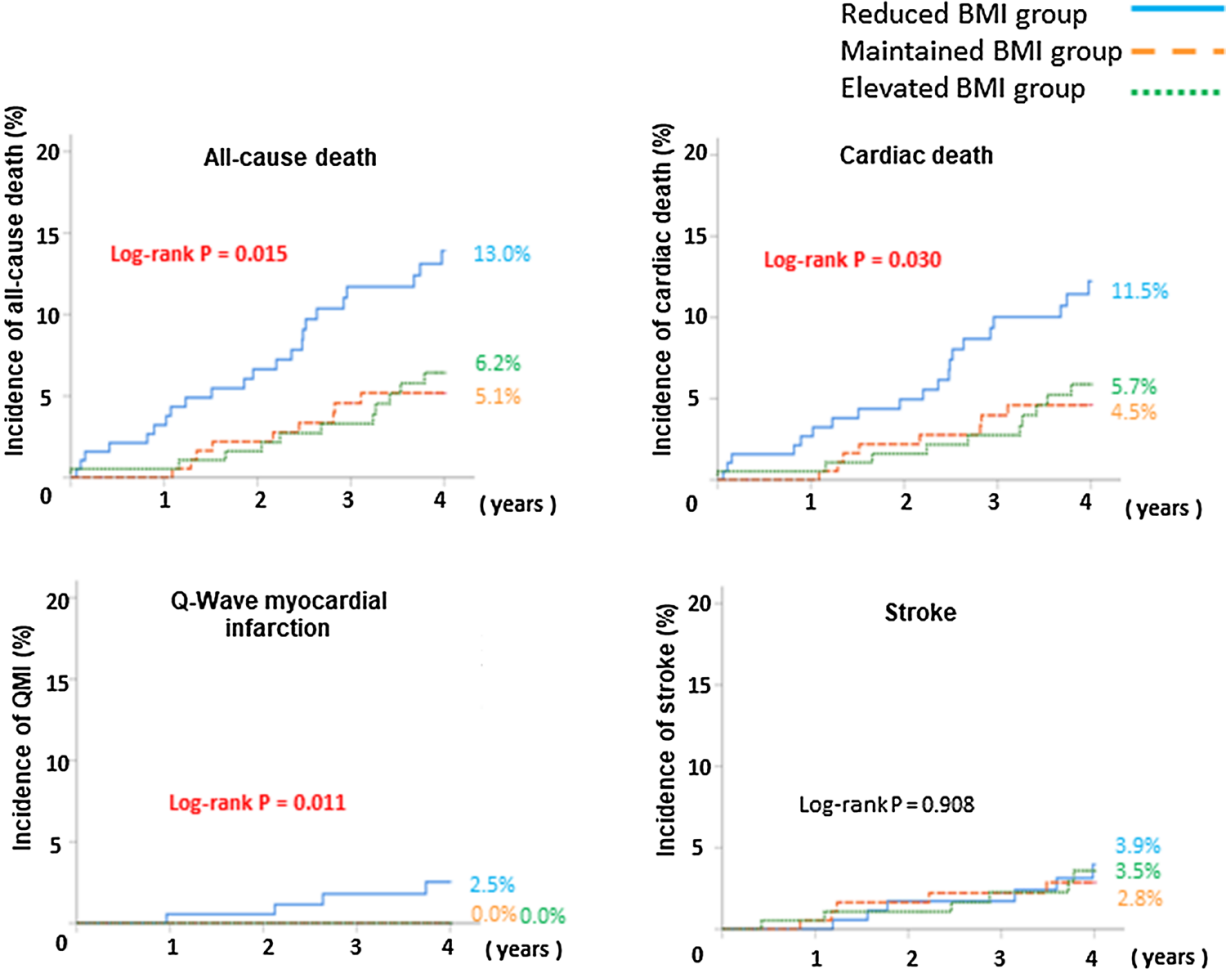
Kaplan–Meier analysis for the incidences of all-cause death, cardiac death, QMI, stroke. Kaplan–Meier curves are shown for the cumulative incidence of all-cause mortality, cardiac death, myocardial infarction, and stroke. The reduced BMI group (< − 0.52 kg/m^2^; *n* = 191) has a significantly higher incidence of all-cause mortality, cardiac death, and myocardial infarction than the maintained BMI group (− 0.52 to 0.38 kg/m^2^; *n* = 191) and elevated BMI group (≥ 0.38 kg/m^2^; *n* = 190). *BMI* body mass index, *QMI* Q-wave myocardial infarction

Figure 2 illustrates the cumulative incidence of MACEs according to each BMI group. The reduced BMI group had a significantly higher incidence of MACEs than the other groups (χ^2^ test for linear trend, *p* = 0.004, respectively). Figure 3 demonstrates the difference in each clinical event, according to change in the BMI tertile. The incidences of all-cause death were significantly higher in patients in the reduced BMI group than in the other groups (*χ*^2^ for linear trend, *p* = 0.015, respectively). This trend was the same for the incidence of cardiac death (*χ*^2^ for linear trend, *p* = 0.030, respectively). Only the reduced BMI group had a Q-wave myocardial infarction. There were no significant differences between the groups in the incidences of stroke (*χ*^2^ for linear trend, *p* = 0.908, respectively). Multivariable Cox regression analysis was performed to identify specific predictors of MACEs in the study population. After adjusting for age, sex, estimated glomerular filtration rate, LV systolic dysfunction, high and low BMI (at the time of registration), and history of hypertension, dyslipidemia, diabetes mellitus, atrial fibrillation, and ACS, reduced BMI showed a strong association with MACEs (hazard ratio 2.24; 95% confidence interval 1.34–3.75; *p* = 0.003) (Table 3).

**Table 3:** Univariate and multivariable predictors of major adverse cardiac or cerebrovascular events

Variable	Unadjusted HR (95% CI)	*p* value	Adjusted HR (95% CI)	*p* value
Age	1.069 (1.037–1.101)	< 0.001	1.056 (1.025–1.087)	< 0.001
Sex	1.323 (0.688–2.545)	0.401		
LVD	1.002 (0.431–2.332)	0.996		
HT	1.206 (0.653–2.229)	0.550		
DLp	0.456 (0.274–0.761)	0.003	0.547 (0.326–0.917)	0.022
DM	1.352 (0.812–2.254)	0.246		
Smoking	0.973 (0.579–1.636)	0.973		
Af	1.354 (0.616–2.979)	0.451		
ACS	1.278 (0.770–2.120)	0.342		
High BMI	0.667 (0.167–2.732)	0.574		
Low BMI	3.017 (1.371–6.640)	0.006	2.768 (1.233–6.213)	0.014
Reduced BMI	2.231 (1.345–3.703)	0.002	2.244 (1.342–3.755)	0.003

*ACS* acute coronary syndrome, *Af* atrial fibrillation, *DM* diabetes mellitus, *DLp* dyslipidemia, *High BMI* high body mass index (> 30 kg/m^2^) immediately after percutaneous coronary intervention, *HT* hypertension, *Low BMI* low body mass index (< 18.5 kg/m^2^) immediately after percutaneous coronary intervention, *LVD* left ventricular dysfunction (left ventricular ejection fraction ≤ 40%), *reduced BMI* reduced body mass index (< − 0.52 kg/m^2^), *CI* confidence interval, *HR* hazard ratio

## Discussion

In this study, we demonstrated that BMI change within 1 year after undergoing PCI could predict MACEs in patients with CAD. In particular, patients with reduced BMI (≥ 0.52 kg/m^2^) had a higher incidence of MACEs than those with a maintained BMI (− 0.52 to 0.38 kg/m^2^) and elevated BMI (≥ 0.38 kg/m^2^) in this study.

Obesity is an independent risk factor for cardiovascular disease, including CAD and heart failure, and is associated with morbidity [4,^ 8^–10]. It has been demonstrated that the relationship between BMI and mortality in elderly patients is represented by a large flat-bottomed U-shaped curve [11]. Additionally, low BMI is independently associated with an increased risk of 1-year mortality after PCI [12]. However, this study showed that weight loss after PCI treatment has impact on prognosis regardless of the weight before PCI treatment. The original mean BMI was normal in each group, and there was no significant difference among groups. The BMI after the 1-year follow-up was also in the normal range in each group.

Although it is unknown in the present study whether patients intentionally lost weight, some studies reported that observational weight loss is associated with increased adverse cardiovascular events, whereas intentional weight loss is associated with decreased clinical events [13]. It is necessary to pay attention to unexpected weight loss in patients with CAD after PCI.

Several reasons for the worsening of prognosis due to weight loss have been reported. One of the causes of poor prognosis due to low body weight is the rapid progression of cachexia. In cachectic patients, skeletal muscle and adipose tissue are reduced [14, 15]. Skeletal muscle loss increases the risk of adverse outcomes, including physical disability, poor quality of life, and death [16]. A study reported that myonectin secreted from skeletal muscle suppresses apoptosis and inflammation in the heart, suggesting that myonectin mediates some of the beneficial actions of the cardiovascular system [17]. In adipose tissue, adiponectin suppresses arteriosclerosis and improves insulin resistance [18]. Thin patients with heart disease have high tumor necrosis factor (TNF)α levels [19]. TNFα is an indicator of systemic inflammation and a poor prognostic factor in patients with heart disease [19, 20]. Obese people have low peripheral vascular resistance, whereas thin people have high vascular resistance. Systemic vascular resistance is associated with the severity of cardiovascular disease [21]. Although it is not clear why weight loss after PCI affected poor prognosis in this study, these acute physical stresses due to short-term weight loss are thought to exacerbate the prognosis after PCI.

The patients in the present study were mainly elderly people (70.2 ± 10.3 years), and aging diseases can cause weight loss. It has been pointed out that aging itself leads to a decrease in immune response and that thin elderly people may be less resistant to infection [22]. Reduced immunity due to weight loss is thought to affect total death.

Obesity is often evaluated using BMI, which is the simplest tool. However, BMI cannot measure fat and muscle mass, nor can it measure visceral fat. Patients with more fat and less muscle mass have a worse prognosis after PCI [23]. Further, the higher the proportion of visceral fat, the higher the risk of CAD [24]. However, it is difficult to measure muscle mass and visceral fat mass in terms of burdens, such as time and cost, in routine examinations. In that respect, BMI can be easily measured in any situation.

In this study, physical activity and cardiorespiratory fitness (CRF) are not evaluated. Some studies show that sustained physical activity and CRF are associated with substantial mortality risk reduction than BMI. Individuals with CAD are recommended to be physically active [25] and to improve their CRF [26, 27]. Furthermore, changes in physical activity had lower all-cause mortality risk than changes in BMI [28]. However, a comparison of changes in physical activity is sometimes difficult to evaluate by an interview, and the measurement of CRF is also difficult in terms of costs than BMI.

In the heart failure era, obesity is a risk factor of developing heart failure; however, obesity works to prevent exacerbation of heart failure in patients who are being treated for chronic heart failure after onset, and low weight is a risk factor for exacerbation of heart failure, i.e., the obesity paradox [25]. Even in the ischemic region, the approach to weight management before and after PCI may change. Although high BMI is a risk factor of developing ischemic events, this study suggests that weight gain after PCI is not a poor prognostic factor, and weight loss after PCI may be a poor prognostic factor. On the other hand, compared to the maintained BMI group, elevated BMI group also shows a little worse outcome, even not statistically significant. In the future, it will be necessary to conduct a positive study with a bigger number of data on the effect of weight change after PCI on prognosis.

### Limitations

This study has several limitations. First, there is possible observer bias because this study was designed as an investigator-driven observational study. Second, in this study, we did not know whether the weight loss was intentional. Third, because the bodyweight cannot be measured after PCI, the sample size was small. Fourth, external monitoring of this registry was lacking. Lastly, there is no consensus on the follow-up of weight changes and outcomes and needs to be considered.

## Conclusions

The long-term clinical outcomes of patients with CAD who underwent PCI were affected by the reduction in BMI after PCI. Furthermore, elevation of BMI after PCI was not a poor prognostic factor. The current study demonstrated that stratification using a change in BMI after PCI may help to predict adverse events in patients with CAD. Long-term studies in larger populations are needed to assess the clinical validation of changes in BMI after PCI with regard to long-term outcomes and in the general population.
